# Vitamin A to prevent bronchopulmonary dysplasia in extremely low birth weight infants: a systematic review and meta-analysis

**DOI:** 10.1371/journal.pone.0207730

**Published:** 2018-11-29

**Authors:** Shunsuke Araki, Shin Kato, Fumihiko Namba, Erika Ota

**Affiliations:** 1 Department of Pediatrics, School of Medicine, University of Occupational and Environmental Health, Yahatanishi, Kitakyushu, Japan; 2 Department of Pediatrics and Neonatology, Nagoya City University Graduate School of Medical Science, Mizuho-ku, Nagoya, Japan; 3 Department of Pediatrics, Saitama Medical Center, Saitama Medical University, Kawagoe, Saitama, Japan; 4 Global Health Nursing, Graduate School of Nursing Science, St. Luke’s International University, Chuo-ku, Tokyo, Japan; Center of Pediatrics, GERMANY

## Abstract

**Background:**

Vitamin A (VA) supplementation reduces the risk of developing bronchopulmonary dysplasia (BPD). However, a previous meta-analysis showed that VA had minimal efficacy for preventing BPD in very low birth weight infants (VLBWIs).

**Aims:**

To elucidate the effects of VA supplementation for BPD prevention in extremely low birth weight infants (ELBWIs).

**Study design:**

This systematic review and meta-analysis followed the Preferred Reporting Items for Systematic Reviews and Meta-Analyses (PRISMA) guidelines. We registered the protocol on PROSPERO, the international prospective registry of systematic reviews (registration number: CRD42016050887). We searched the following five databases: CINAHL, CENTRAL, EMBASE, MEDLINE, and PubMed; screened the reference lists of retrieved articles to identify randomized controlled trials (RCTs); and assessed the Cochrane Risk of Bias for each study. The certainty of the evidence was assessed using the Grading of Recommendations Assessment, Development and Evaluation (GRADE) guidelines.

**Results:**

Four studies (total, 1,011 infants) were included. VA was administered intramuscularly in 3 studies and orally in 1 study. VA supplementation for ELBWIs had benefited oxygen dependency at the postmenstrual age of 36 weeks in survivors (pooled risk ratio, 0.88; 95% confidence intervals (CI), 0.77–0.99; 4 trials, 841 infants, moderate certainty of evidence), which is similar to the meta-analysis in VLBWIs. Length of hospital stay was reduced in the VA group (mean difference, −49.9; 95% CI, −88.78 to −11.02; 1 trial, 20 infants, low certainty of evidence). The meta-analysis showed no reduction in the risk of neonatal death, oxygen use at 28 days in survivors, duration of mechanical ventilation, intraventricular hemorrhage, retinopathy in prematurity, and necrotizing enterocolitis.

**Conclusions:**

VA supplementation for ELBWIs is potentially effective in decreasing oxygen dependency at the postmenstrual age of 36 weeks.

## Introduction

Vitamin A plays an important role in the growth, differentiation, and maintenance of airway epithelial cells [1, 2] and has been an effective treatment intervention for premature infants suffering from the major respiratory sequel bronchopulmonary dysplasia (BPD). In premature infants, vitamin A levels are insufficient at birth because the accumulation of vitamin A in the fetus occurs mainly in the third trimester [3]. Researchers have compared plasma vitamin A levels in early infancy with BPD prognoses and found that lower vitamin A concentration is associated with the development of BPD [4]. To improve respiratory prognosis, different doses of vitamin A and methods of delivery, such as intramuscular injection, oral supplementation, and intravenous infusion, have been tested in clinical trials [5–10]. A recent Cochrane systematic review focusing on very low birth weight infants (VLBWIs, <1,500 g) found that vitamin A supplementation had a small benefit on the risk of decreased death and on oxygen dependency at 28 days and resulted in a marginal reduction in oxygen use at the postmenstrual age of 36 weeks [11].

Although vitamin A is a potentially effective nutrient for the prevention of BPD, it has not been incorporated into standard care because of its relatively small benefits and the need for repeated intramuscular injections. In a clinical trial conducted in Europe, infants were supplemented with oral vitamin A to avoid repeated muscular injections; the trial is still in the process of recruiting patients [10, 12]. Another reason for the sparse use of Vitamin A might be that the largest study on extremely low birth weight infants (ELBWI; <1,000 g) was conducted more than 20 years ago by Tyson et al. [8], which brings into question the applicability of the results given recent advances in respiratory care.

We hypothesized that variability among participating VLBWIs will compromise the results of the analysis and that focusing on ELBWIs will improve the relatively weak results previously observed. The present meta-analysis aimed to find whether vitamin A supplementation in ELBWI reduced risk of death at 28 days and at hospital discharge and/or risk of BPD defined as oxygen use at 28 days (BPD 28) and at the postmenstrual age of 36 weeks (BPD 36).

## Material and methods

We conducted a systematic review of randomized controlled trials (RCTs) using standard methods from the Cochrane Handbook for Systematic Reviews of Intervention. We registered the protocol on PROSPERO, the international prospective registry of systematic reviews (registration number: CRD42016050887). We followed the reporting guidelines outlined in the Preferred Reporting Items for Systematic Reviews and Meta-Analyses (PRISMA) statement [13] (S1 Table).

### Search strategy

We searched five electronic databases, namely, CINAHL, CENTRAL, EMBASE, MEDLINE via Ovid SP, and PubMed on October 31, 2016, with no limitations on date/time, language, document type, or publication status. Keywords were established after expert opinion, literature review, and controlled vocabulary (Medical Subject Headings, Excerpta Medica Tree, and CINAHL headings). The final search terms included “vitamin A or retinol or retinoid or retinoic or aquasol,” “bronchopulmonary dysplasia,” “lung diseases,” “chronic disease,” “infant,” “very low birth weight,” “extremely low birth weight,” and “extremely premature” (S2 Table).

### Identification of studies

We identified RCTs by comparing the effects of vitamin A versus control in premature infants with a birth weight <1,000 g. An information specialist conducted the initial searches, and the reviewers manually conducted supplemental searches. Regarding unpublished trials, the authors were contacted for further information. Two of the authors of this review independently assessed all studies identified for further review using Rayyan systematic reviews web application (http://rayyan.qcri.org/). Differences in opinion of the assessment were resolved by personal discussions or by obtaining the opinion of a third evaluator. Two of the reviewers used piloted data extraction forms to collect basic study information and details on participants, number of ELBWIs, treatment and control interventions, and outcome data; Death at 28 days and at hospital discharge, Oxygen use at 28 days or at 36 weeks’ postmenstrual age, duration of ventilation, length of stay at hospital, morbidity of intraventricular hemorrhage, retinopathy of prematurity, necrotizing enterocolitis and occurrence of adverse effects (S3 Table). BPD was defined as the need for oxygen at 36 week’s postmenstrual age in all 4 studies.

### Data analysis

Data were analyzed using Review Manager 5 software (RevMan 5, 2014 http://community.cochrane.org/tools/review-production-tools/revman-5). Risk ratios (RR) were used for dichotomous data, and the weighted mean difference or standardized mean difference (MD) was used for continuous data. Results of a fixed effects analysis are presented as average intervention effect with 95% confidence intervals (CIs). Statistical heterogeneity was estimated by the I^2^ test. A value of I^2^ >75% is considered substantial heterogeneity and 30% to 60% may represent moderate heterogeneity. If there were only a few studies or if the sample size was small, we used a fixed-effect model, since random-effects models provide poor estimates of the intervention effects distribution.

### Assessment of risk of bias in included studies

Two of the authors independently assessed the risk of bias in the studies included in this meta-analysis using methods from the Cochrane Handbook for Systematic Reviews of Interventions. Differences in opinion were resolved by discussion between the authors or obtaining an opinion from a third evaluator.

### Quality of evidence

To rate the certainty of evidence, we used the summary of findings template in the Guideline Development Tool developed by the Grading of Recommendations, Assessment, Development and Evaluation (GRADE) Working Group (http://www.gradeworkinggroup.org). The studies were evaluated in compliance with GRADE, and the rating of the evidence was determined according to GRADE’s five downgraded criteria: risk of bias, inconsistency, indirectness, imprecision, and publication bias [14]. A certainty rating was given for each of the following outcome: neonatal death; oxygen use at 28 days in survivors, and BPD at the postmenstrual age of 36 weeks in survivors. These ratings were classified according to the following four levels of certainty recommended by the GRADE approach: high, moderate, low, and very low certainty of evidence.

## Results

### Search results

**Fig 1:**
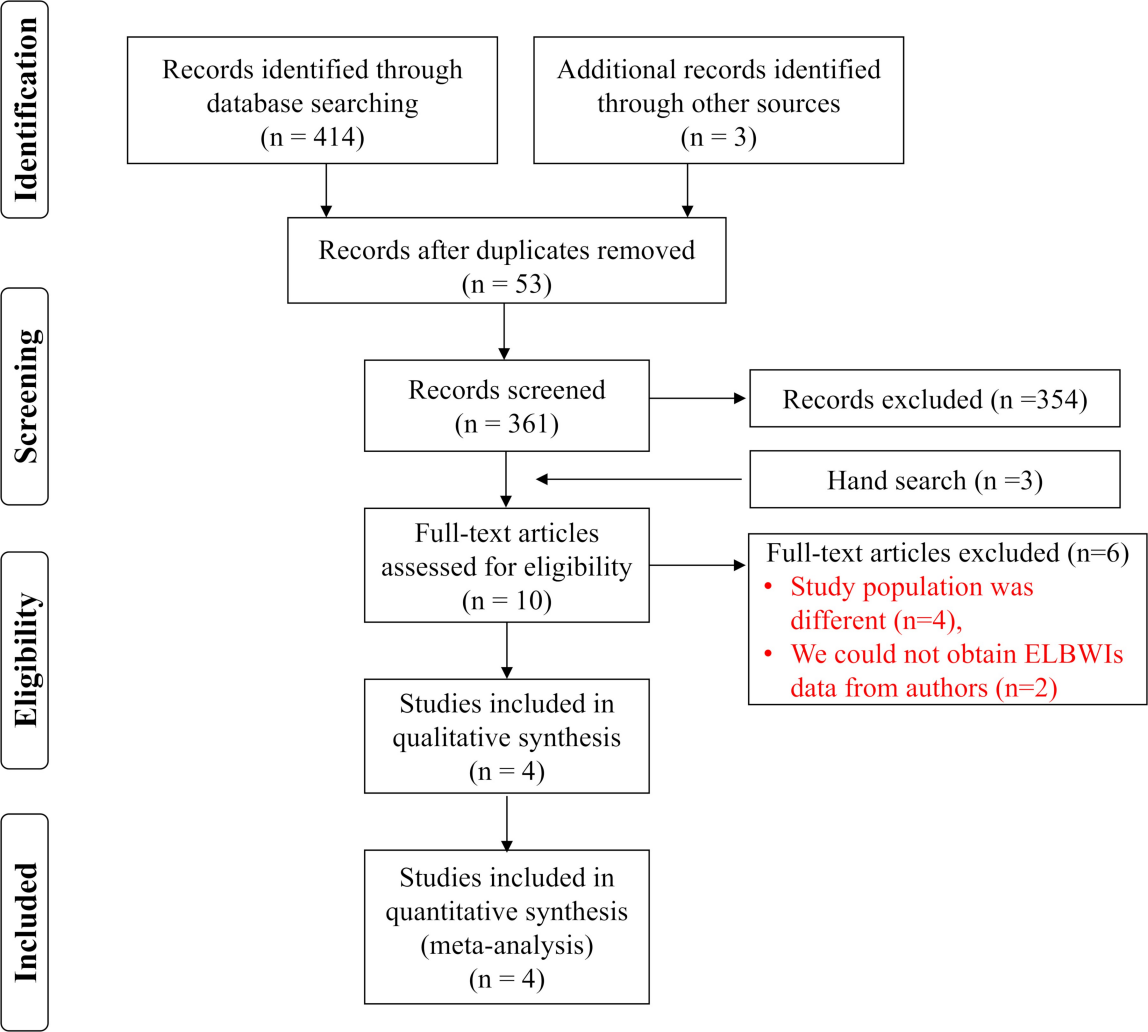
Flow diagram of search results and study selection.

The initial search identified 414 reports through database searching. Of these, 53 duplicated reports were removed, and 361 reports were screened. Seven reports (Papagaroufalis 1988 [15] (unpublished data only), Calisici 2014 [16], Pearson 1992 [17], Shenai 1992 [18], Tyson 1999 [8], Wardle 2001 [9], and Kiatchoosakun 2014 [5]) met the inclusion criteria, and we also added three hand-searched reports (Mactier 2012 [6], Ravishankar 2013 [7], and Werkman 1994 [19]). Of them, we excluded six reports because the study population was different or because we could not obtain data on ELBWIs from authors (Fig 1).

### Included studies

Four studies (Tyson 1999 [8], Wardle 2001 [9], Mactier 2012 [6] and Kiatchoosakun 2014 [5] (total 1,011 ELBWIs) were included in this review, and all studies compared the effects of vitamin A versus control on neonatal outcomes. However, some differences existed between the studies in terms of participants, settings, and interventions (i.e., route, dose, and timing of vitamin A supplementation). VA was administered intramuscularly in 3 studies and orally in 1 study (Wardle 2001). In addition, there were differences in the outcomes of interest and the methods used to measure them. Not all studies reported results that addressed our primary outcomes (i.e., neonatal mortality, and BPD at 28 days and at the postmenstrual age of 36 weeks in survivors) and for some outcomes, not all studies provided data.

### Participants

All participants in the studies included were ELBWIs. The Kiatchoosakun 2014 study included 80 VLBWIs, which in turn included 20 ELBWIs, who received mechanical ventilation or oxygen supplementation 24 hours after birth. The Tyson 1999 study included 807 ELBWIs weighing 401–1,000 g who required supplemental oxygen or mechanical ventilation 24 hours after birth. In Wardle 2001, 154 ELBWIs were included. The Mactier 2012 study comprised 89 infants, including ELBWIs, who were born <32 weeks of gestation, had a birth weight <1,501 g, or both.

### Locations

All studies included were conducted in the United States (Tyson 1999), the United Kingdom (Wardle 2001 and Mactier 2012), or Thailand (Kiatchoosakun 2014).

### Interventions

In Tyson 1999, a regimen of 5,000 international units (IU) of vitamin A supplement (water-soluble retinyl palmitate) three times per week for 4 weeks by intramuscular injection was compared with a sham injection. In Wardle 2001, 5,000 IU/kg of supplemental vitamin A (form not stated) was administered orally each day till day 28 and was compared with the same volume of a placebo liquid. Infants in both the control and treatment groups also received 23 IU/kg/d vitamin A, added to Intralipid, when they were on parenteral nutrition. In Kiatchoosakun 2014, infants were assigned to receive either 5,000 IU intramuscular vitamin A 3 times per week (treatment group) or a sham procedure (control group) for 4 weeks. In Mactier 2012, supplemental infants received an additional 10,000 IU intramuscular vitamin A 3 times per week, starting from day 2, for a minimum of 2 weeks, or until the establishment of oral feeding.

### Outcomes

We contacted four trial authors for detailed outcome data on ELBWIs and were provided with the detailed results for Kiatchoosakun 2014 and Mactier 2012.

In Tyson 1999, the outcomes were oxygen requirement at or death before the postmenstrual age of 36 weeks, oxygen requirement at or death before 28 days of age, culture-proven sepsis, grade 3 or 4 intraventricular hemorrhage (IVH), periventricular leukomalacia, serum vitamin A concentrations on day 28, and relative dose-response to 2,000 IU vitamin A intramuscular supplementation in a subgroup of infants. The potential toxicity of vitamin A was evaluated weekly.

In Wardle 2001, the outcomes were death pre-discharge, oxygen requirement at the postmenstrual ages of 28 days and 36 weeks, and retinopathy of prematurity (ROP) requiring treatment.

In Kiatchoosakun 2014, the outcomes were serum vitamin A levels and complications of prematurity, which included supplemental oxygen at the postmenstrual age of 36 weeks, duration of intubation, days on oxygen therapy, and length of hospital stay.

In Mactier 2012, hepatic stores of vitamin A were evaluated by relative dose response. The primary outcome was cone-corrected dark-adapted retinal rod sensitivity, which was measured using an electroretinogram at the postmenstrual age of 36 weeks. Reported complications of prematurity included the following: use of respiratory support, IVH, ROP, supplemental oxygen at the postmenstrual age of 36 weeks, and death.

### Risk of bias

**Fig 2:**
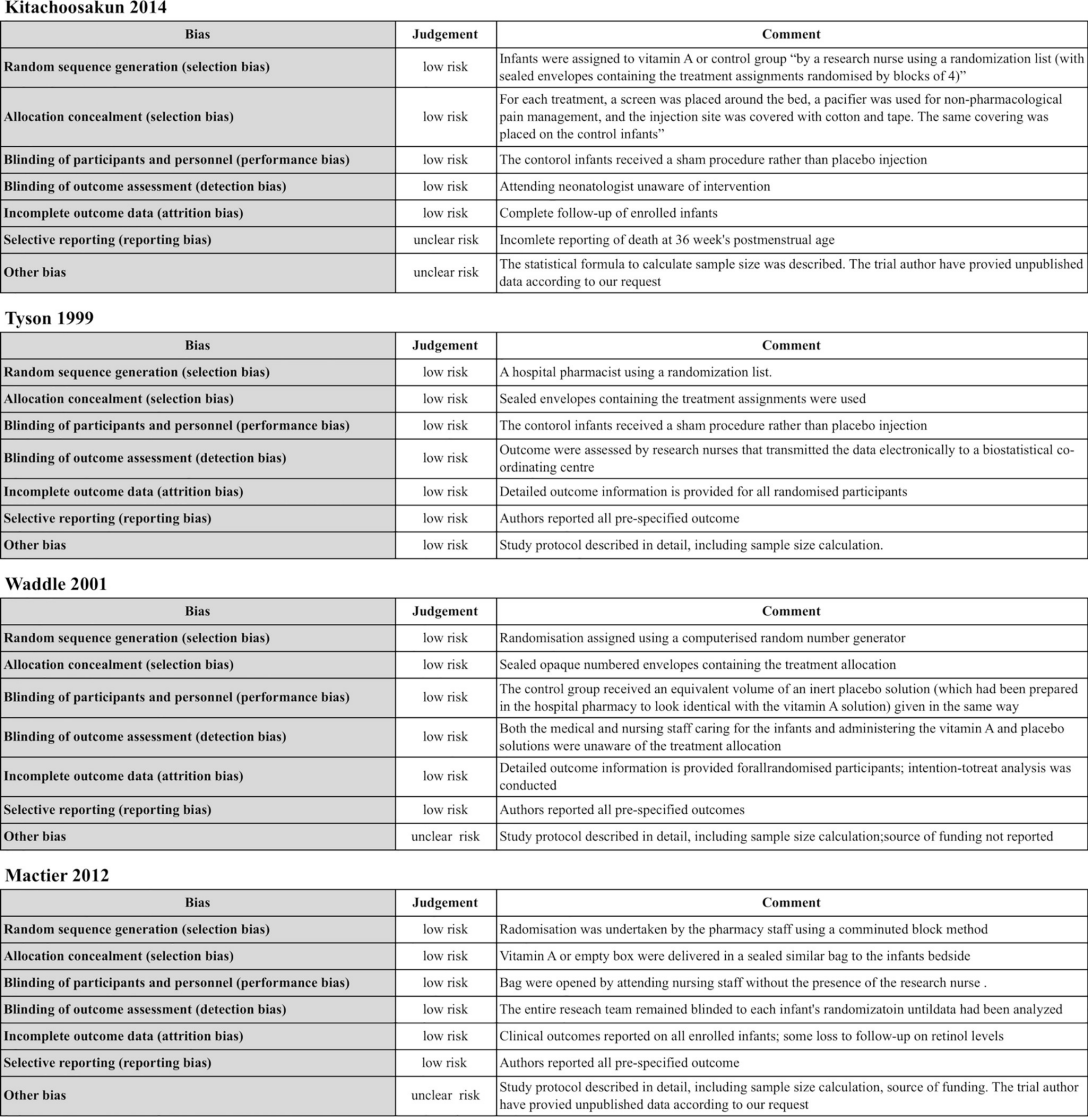
Risk of bias in included studies.

Assessment of the risk of bias assessment of the four studies was conducted (Fig 2).

#### Allocation

The randomization and allocation of the four studies in this review was conducted by someone outside of the trial’s research team using sealed envelopes (low risk of bias).

#### Blinding

All trials participants were blinded to the intervention and outcome variables. Tyson 1999, for example, used sealed envelopes containing the treatment assignments.

#### Incomplete outcome data

All trials included complete follow-up of participants (low risk of bias).

#### Selective reporting

All trials measured prespecified outcomes and reported on all expected outcome measures of interest. However, we used additional data from Mactier 2012 and Kiatchoosakun 2014, which the trial authors provided on request (unclear risk bias).

#### Quality of evidence (GRADE)

**Fig 3:**
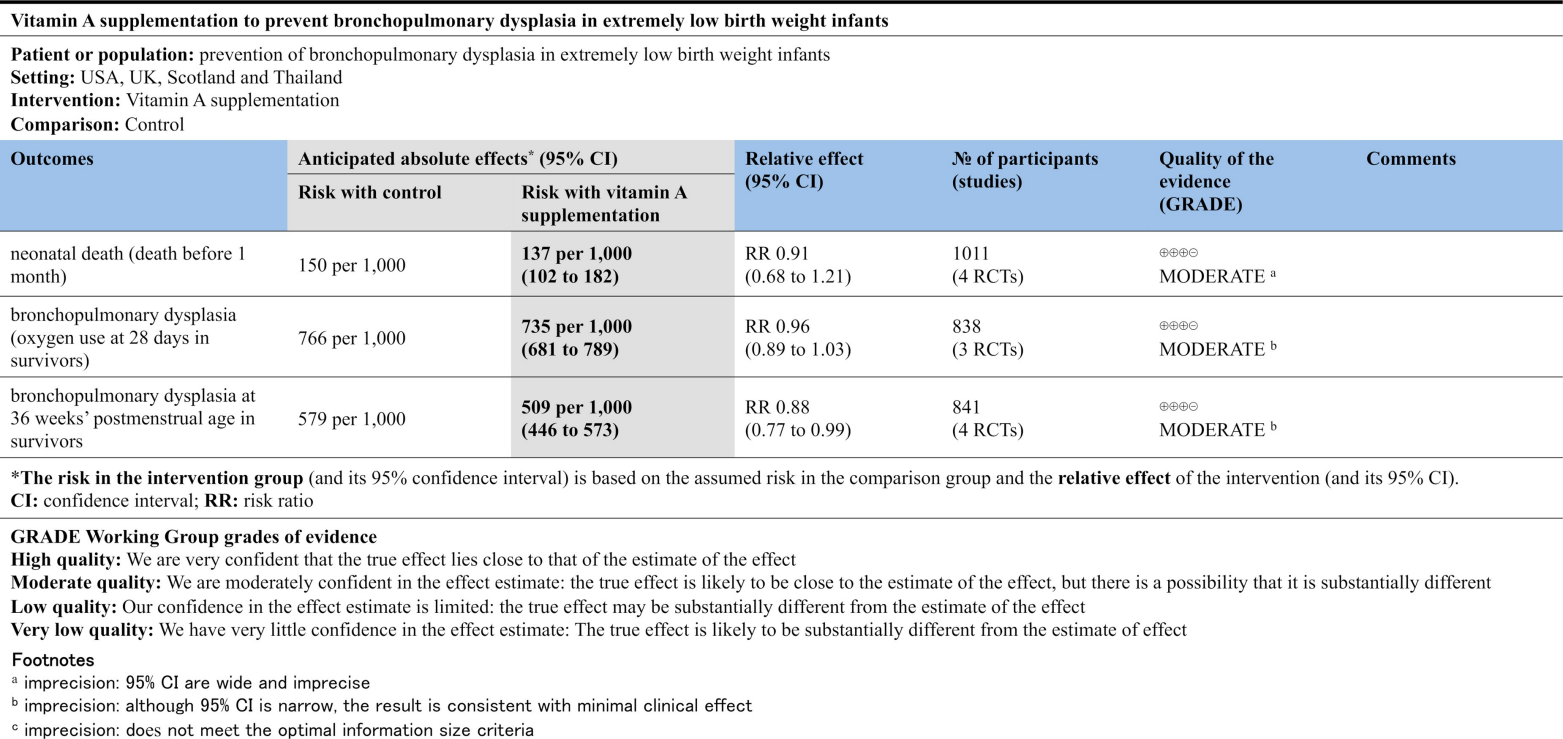
Summary of findings.

The quality of evidence for death at 28 days in the neonatal intensive care unit was rated as moderate and downgraded due to wide 95% CIs. BPD (oxygen use at 28 days and at the postmenstrual age of 36 weeks in survivors) was rated as being of moderate quality of evidence due to minimal clinical effects, although the 95% CI was narrow. A summary of the quality of evidence of the studies in our review is presented in the GRADE summary of findings (Fig 3).

### Effects of the interventions

#### Neonatal death (death before 1 month)

**Fig 4:**
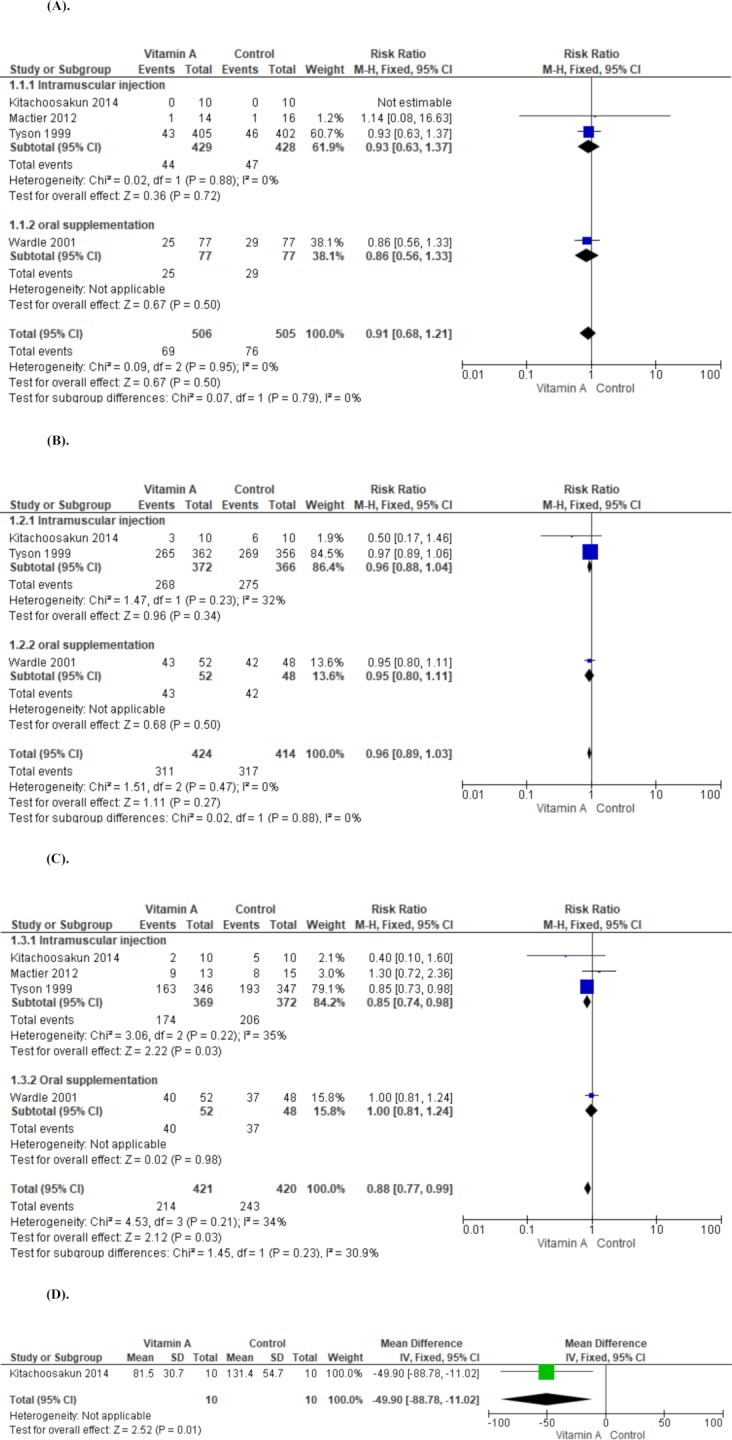
(A). Comparison of vitamin A supplementation versus control. Outcome 1: neonatal death (death before 1 month). (B). Comparison of vitamin A supplementation versus control. Outcome 2: bronchopulmonary dysplasia (oxygen use at 28 days in survivors) (C). Comparison of vitamin A supplementation versus control. Outcome 3: bronchopulmonary dysplasia at 36 weeks’ postmenstrual age in survivors (D). Comparison of vitamin A supplementation versus control. Outcome 4: length of hospital stays.

In the four studies that were included in our review, 69 infants died in the neonatal intensive care unit before discharge. All trials reported on neonatal death and none showed a significant difference between the vitamin A and control groups. The meta-analysis showed no reduction in the risk of neonatal death associated with receiving vitamin A (RR, 0.91; 95% CI, 0.68–1.21; moderate quality of evidence). There was low statistical heterogeneity in the lack of discrepancy between the trials (I^2^ = 0%) (Outcome 1, Fig 4(A)).

#### BPD (oxygen use at 28 days in survivors)

All, except Mactier 2012, reported on BPD at 28 days. Tyson 1999, which was the largest trial (718 infants), showed no significant difference between the vitamin A and control groups (RR, 0.97; 95% CI, 0.89–1.06). Meta-analysis of the three trials showed no reduction in the risk of BPD at 28 days associated with receiving supplemental vitamin A (RR, 0.91; 95% CI, 0.68–1.21, moderate quality of evidence) (Outcome 2, Fig 4(B)).

#### BPD at the postmenstrual age of 36 weeks in survivors

All four trials reported on this outcome. Tyson 1999 reported a significant reduction and Kiatchoosakun 2014 reported a trend in reduction (RR, 0.40; 95% CI, 0.10–1.60) in BPD at the postmenstrual age of 36 weeks. The meta-analysis supported a reduction in the risk of BPD at the postmenstrual age of 36 weeks in the vitamin A treatment group (RR, 0.88; 95% CI, 0.77–0.99; moderate quality of evidence). This outcome showed moderate heterogeneity (I^2^ = 30.9%) (Outcome 3, Fig 4(C)).

#### Length of hospital stay

Two trials reported on this outcome (Tyson 1999 and Kiatchoosakun 2014). Kiatchoosakun demonstrated a significant reduction in duration of hospital stay in the vitamin A treatment groups [mean difference (MD), −49.9; 95% CI −88.78 to −11.02; 20 infants]. The study by Tyson et al. did not report the mean value and standard deviation; therefore, we have excluded these results from the analysis (Outcome 4, Fig 4(D)).

#### Duration of mechanical ventilation

Three trials, with a total of 101 infants, measured this outcome (Kiatchoosakun 2014, Mactier 2012, and Wardle 2001). No significant difference was observed between the vitamin A and control groups (MD, −1.35; 95% CI, −11.38 to 8.68; moderate quality of evidence). There was low statistical heterogeneity in the lack of discrepancy between the trials (I^2^ = 0%) (Outcome 5, S1 Fig).

#### IVH

All the included studies reported on any grade of IVH for a total of 177 infants. No significant difference was observed between the vitamin A group and control (RR, 0.90; 95% CI, 0.77–1.06; moderate quality of evidence). There was low statistical heterogeneity in the lack of discrepancy between the trials (I^2^ = 0%) (Outcome 6, S2 Fig).

#### ROP

Three of the studies included reported on this outcome for a total of 101 infants (Wardle 2001, Mactier 2012, and Kitachoosakun 2014). No significant difference was observed between the vitamin A group and control (RR, 1.40; 95% CI 0.77–2.55; moderate quality of evidence). There was low statistical heterogeneity in the lack of discrepancy between the trials (I^2^ = 0%) (Outcome 7, S3 Fig).

#### Necrotizing enterocolitis (NEC)

Three trials reported on NEC (Tyson 1999, Wardle 2001, and Kiatchoosakun 2014). The meta-analysis showed no significant difference between the vitamin A and control groups (RR, 0.95; 95% CI, 0.69–1.32; 506 infants) (Outcome 8, S4 Fig).

#### Adverse effects

Wardle 2001 reported that there were no potential adverse effects associated with Vitamin A, such as seizures or persistent vomiting, in their study. Tyson 1999 reported conducting weekly assessments for signs of toxicity. Suspected or definite increase in fontanel tension was slightly more frequent in the control group compared with the vitamin A group (18% versus 15%, *p* = 0.26). They also reported potential toxicity unexplained by other factor as 0.8% in the control group and 1.0% in the vitamin A group. Kiatchoosakun 2014 reported no signs of potential vitamin A toxicity in their study and Mactier 2012 did not mention any adverse effects.

## Discussion

This review included ELBWIs who were at high risk of developing BPD. We found that, in RCTs, vitamin A supplementation in ELBWIs has shown a small benefit for reducing oxygen dependency at 36 weeks of corrected gestational age. Statistical significance between vitamin A supplemented and control groups was not confirmed for neonatal death before 1 month; oxygen dependency at 28 days of age; duration of mechanical ventilation; death or neurodevelopmental impairment at 18–22 months; and any grade of IVH, ROP, and NEC.

Clinical trials on the effect of vitamin A supplementation on premature infants have shown relatively mild benefits [5–9, 18]. However, the birth weights of infants participating in those clinical trials were in the range of 401–1,499 g. In fact, VLBWIs were recruited in the original reports (Kiatchoosakun 2014 and Mactier 2012) included in this analysis (S3 Table). This wide-ranging population may have skewed their results because prematurity is a major modifying factor in clinical courses such as incidence of BPD and other major morbidities. Based on this, we hypothesized that focusing on ELBWIs as participants may lead to a much clearer analysis of the benefits of vitamin A supplementation in the prevention of BPD.

In a study on ELBWIs, Tyson [8] reported decreases in death or BPD at the postmenstrual age of 36 weeks associated with intramuscular 5,000 IU injections, 3 times per week for 4 weeks. However, the CI of the result was wide. In addition, the study was conducted more than 20 years ago, so the results may not still be applicable. Tolia et al. [20] reported in their retrospective survey that the incidence of death or BPD before hospital discharge did not change even after a national shortage in injectable vitamin A. Less invasive approaches to perinatal respiratory management, such as the use of noninvasive respiratory support, perinatal corticosteroid, caffeine, and high flow nasal cannula may decrease the incidence of BPD and cancel out the relatively mild effect of vitamin A.

Vitamin A reportedly plays a critical role in alveolar epithelial cell growth, differentiation, development, and maintenance [1, 2] as well as lung injury prevention in animal models, [21] and it is thus used to prevent BPD in premature babies [8, 9, 17, 18]. ELBWIs are at high risk of vitamin A deficiency because of its low stores at birth [22], a tendency for inadequate enteral and parenteral intake after birth, and premature enteral absorption [8, 23, 24]. Tyson reported that serum retinol concentration on day 28 was low (<200 μg/L) in 73% of ELBWIs who received a sham procedure (control group) and that 20% of participants were found to have <100 μg/L of Vitamin A [8], which indicates deficiency. Because of ELBWI’s natural vitamin A shortage and lower absorption efficiency, vitamin A supplementation may be beneficial for reducing their risk of developing BPD.

Some trials employed oral vitamin A supplementation to take advantage of a less invasive route compared with intramuscular injections [9, 10].Due to participants’ underdeveloped and unclear enteral capacity to absorb vitamin A, the researchers tried various supplementation regimens. Wardle [9] measured serum vitamin A concentration at 24 hours after the first dose and on days 7 and 28, with a daily oral dose of 5,000 IU/kg. Vitamin A concentration was significantly higher only at 24 hours after the first dose in supplemented infants (230 μg/L versus 150 μg/L). At 7 and 28 days of age, the median concentration of vitamin A in both groups was >200 μg/L. Currently, there is insufficient data about oral vitamin A supplementation. One area of uncertainty concerns the methods (e.g., timing of intake and number of doses per day) necessary to maintain serum vitamin A levels. Others are the bioavailability of vitamin A and whether oral supplementation can reduce the incidence of BPD in ELBWIs. Further research is needed to elucidate a minimum effective regimen to prevent BPD and avoid overdosing.

A larger phase III RCT has been launched, and eligible newborns are being registered [10, 12]. In this trial, the researchers focused on ELBWIs and employed oral vitamin A supplementation with basic 1,000 IU/kg/day in all infants and an additional 5,000 IU/kg/day in the intervention group for 28 days. Because of the need for repeated intramuscular vitamin A administration and the related pain, oral vitamin A supplementation may provide a good alternative.

Although the findings of our meta-analysis support the benefits of vitamin A for ELBWIs, they should be interpreted in the context of the study’s limitations. First, we were able to include only four studies even after contacting authors to request information about ELBWIs. To support our findings, clinical trials having a larger sample size are necessary, and generalization of our results should be considered cautiously. Second, our results are relatively weak due to the heterogeneity in the trials introduced by different dosing protocols and modes of drug delivery. Including trials that are currently in progress may improve the strength of the analysis. Third, the biggest study, by Tyson et al., was conducted decades ago, and recent advances in the management of respiratory care in ELBWIs may lead to different results even when applying the same protocol.

Another systematic review about vitamin A supplementation for BPD in ELBWIs was published very recently [25]. Those results strongly agreed with ours, including a reduction in the incidence of BPD and combined outcomes of mortality/BPD at the postmenstrual age of 36 weeks. However, despite that study’s literature search for RCTs through 2 January 2018, only two older RCTs (Tyson 1999 [8] and Wardle 2001 [9]) were included; the two newer RCTs (Kiatchoosakun 2014 [5] and Mactier 2012 [6]) were not included.

## Conclusions

Vitamin A supplementation in ELBWIs is a potentially effective intervention to decrease oxygen dependency at the postmenstrual age of 36 weeks. This systematic review included studies employing enteral and parenteral application of vitamin A, resulting in limitations for the conclusions. While intramuscular injection of vitamin A has been reported to be effective in VLBWIs, further research-based evidence on the prevention of BPD using less invasive oral supplementation is needed. Detailed data on pharmacokinetics, accumulation, and the efficiency of using vitamin A in ELBWIs, particularly via oral supplementation, is also necessary. Further clinical trials are needed to determine the sufficient amount for supplementation and the most effective method for delivery.

## Supporting information

S1 Table PRISMA 2009 checklist. (DOC)

S2 Table The full search strategies. (DOCX)

S3 Table Characteristics of included studies. (PPTX)

S1 Fig Comparison of vitamin A supplementation versus control. Outcome 5: duration of mechanical ventilation.
(PPTX)

S2 Fig Comparison of vitamin A supplementation versus control. Outcome 6: any grade of intraventricular hemorrhage.
(PPTX)

S3 Fig Comparison of vitamin A supplementation versus control. Outcome 7: retinopathy of prematurity.
(PPTX)

S4 Fig Comparison of vitamin A supplementation versus control. Outcome 8: necrotizing enterocolitis.
(PPTX)
