# Supplementary material for: Vitamin A to prevent bronchopulmonary dysplasia in extremely low birth weight infants: a systematic review and meta-analysis
Source: PLoS One. 2018 Nov 29;13(11):e0207730. doi: 10.1371/journal.pone.0207730 (PMC6264498; doi:10.1371/journal.pone.0207730)
Supplement: S2 Table — (DOCX) [file pone.0207730.s002.docx]

**S2 Table Search strategies**

**A: Ovid MEDLINE(R) In-Process & Other Non-Indexed Citations and Ovid MEDLINE(R) <1946 to Present>**

1 exp Vitamin A/ (45174)

2 (vitamin A or retinol or retinoid or retinoic or aquasol).tw,nm. (76945)

3 or/1-2 (80398)

4 exp Bronchopulmonary Dysplasia/ (4086)

5 exp Lung Diseases/ (828594)

6 exp Chronic Disease/ (255635)

7 (bronchopulmonary dysplasia or lung disease$ or lung injur$).tw. (72389)

8 or/4-7 (1078889)

9 Infant, Very Low Birth Weight/ (7791)

10 Infant, Extremely Low Birth Weight/ (1676)

11 Infant, Extremely Premature/ (1140)

12 (Neonate or Neonatal or Extremely premature or Extremely preterm or Extremely low birth weight or very low birth weight or low birth weight or LBW or VLBW or ELBW).tw. (218768)

13 or/9-12 (221340)

14 3 and 8 and 13 (122)

15 exp clinical trial/ (816569)

16 exp randomized controlled trials/ (121318)

17 exp double-blind method/ (147658)

18 exp single-blind method/ (24527)

19 exp cross-over studies/ (42567)

20 randomized controlled trial.pt. (469524)

21 clinical trial.pt. (527674)

22 controlled clinical trial.pt. (95062)

23 (clinic$ adj2 trial).mp. (679641)

24 (random$ adj5 control$ adj5 trial$).mp. (641670)

25 (crossover or cross-over).mp. (84643)

26 ((singl$ or double$ or trebl$ or tripl$) adj (blind$ or mask$)).mp. (213881)

27 randomi$.mp. (764423)

28 (random$ adj5 (assign$ or allocat$ or assort$ or reciev$)).mp. (213680)

29 or/15-28 (1267664)

30 Epidemiologic studies/ (7947)

31 exp case control studies/ (876201)

32 exp cohort studies/ (1712991)

33 Case control.tw. (108175)

34 (cohort adj (study or studies)).tw. (141334)

35 Cohort analy$.tw. (5732)

36 (Follow up adj (study or studies)).tw. (44854)

37 (observational adj (study or studies)).tw. (71121)

38 Longitudinal.tw. (199339)

39 Retrospective.tw. (380172)

40 Cross sectional.tw. (255288)

41 Cross-sectional studies/ (254668)

42 or/30-41 (2482664)

43 29 or 42 (3454338)

44 14 and 43 (49) Obs and RCTS

45 14 and 29 (27) RCTS only

**B: Embase <1974 to 2016 November 22>**

1 exp retinol/ (42008)

2 (vitamin A or retinol or retinoid or retinoic or aquasol).tw,tn. (69832)

3 or/1-2 (88464)

4 exp lung dysplasia/ (9366)

5 exp lung disease/ (1127176)

6 exp chronic lung disease/ (10925)

7 (bronchopulmonary dysplasia or lung disease$ or lung injur$).tw. (96040)

8 or/4-7 (1135968)

9 very low birth weight/ (11041)

10 extremely low birth weight/ (3048)

11 (Neonate or Neonatal or Extremely premature or Extremely preterm or Extremely low birth weight or very low birth weight or low birth weight or LBW or VLBW or ELBW).tw. (260576)

12 or/9-11 (263044)

13 3 and 8 and 12 (280)

14 (clin$ adj2 trial).mp. (1286273)

15 ((singl$ or doubl$ or trebl$ or tripl$) adj (blind$ or mask$)).mp. (251298)

16 (random$ adj5 (assign$ or allocat$)).mp. (147417)

17 randomi$.mp. (923667)

18 crossover.mp. (80888)

19 exp randomized-controlled-trial/ (463560)

20 exp double-blind-procedure/ (138126)

21 exp crossover-procedure/ (53903)

22 exp single-blind-procedure/ (27421)

23 exp randomization/ (83727)

24 or/14-23 (1835220)

25 Clinical study/ (255152)

26 Case control study/ (122809)

27 Family study/ (28225)

28 Longitudinal study/ (105753)

29 Retrospective study/ (515332)

30 Prospective study/ (387082)

31 Randomized controlled trials/ (124977)

32 30 not 31 (382341)

33 Cohort analysis/ (301736)

34 (Cohort adj (study or studies)).tw. (179369)

35 (Case control adj (study or studies)).tw. (100754)

36 (follow up adj (study or studies)).tw. (53634)

37 (observational adj (study or studies)).tw. (101326)

38 (epidemiologic$ adj (study or studies)).tw. (89033)

39 (cross sectional adj (study or studies)).tw. (131624)

40 or/25-29,32-39 (1847380)

41 24 or 40 (3409966)

42 13 and 41 (128) obs and RCTS

43 13 and 24 (101) RCTS only

**C: Cochrane Library**

#1 MeSH descriptor: [Vitamin A] explode all trees [1686]

#2 (vitamin A or retinol or retinoid or retinoic or aquasol):ti,ab,kw (Word variations have been searched)[16353]

#3 #1 or #2 [16587]

#4 MeSH descriptor: [Bronchopulmonary Dysplasia] explode all trees [350]

#5 MeSH descriptor: [Lung Diseases] explode all trees [30536]

#6 MeSH descriptor: [Chronic Disease] explode all trees [12115]

#7 (bronchopulmonary dysplasia or lung disease* or lung injur*):ti,ab,kw (Word variations have been searched) [18371]

#8 #4 or #5 or #6 or #7 [51640]

#9 MeSH descriptor: [Infant, Very Low Birth Weight] explode all trees [841]

#10 MeSH descriptor: [Infant, Extremely Low Birth Weight] explode all trees [99]

#11 MeSH descriptor: [Infant, Extremely Premature] explode all trees [80]

#12 (Neonate or Neonatal or Extremely premature or Extremely preterm or Extremely low birth weight or very low birth weight or low birth weight or LBW or VLBW or ELBW):ti,ab,kw (Word variations have been searched) [15893]

#13 #9 or #10 or #11 or #12 [15893]

#14 #3 and #8 and #13 in Trials [46] RCTS only

**D: CINAHL**

S35 S12 AND S24 AND S30 AND S33 RCT only (233)

S34 S21 AND S24 AND S30 AND S33 Obs and RCTS (280)

S33 S31 OR S32 (97,337)

S32 TX (Neonate or Neonatal or "Extremely premature" or "Extremely preterm" or "Extremely low birth weight" or "very low birth weight" or "low birth weight" or LBW or VLBW or ELBW) (97,337)

S31 (MH "Infant, Very Low Birth Weight") (3,621)

S30 S25 OR S26 OR S27 OR S28 OR S29 (174,809)

S29 TX "bronchopulmonary dysplasia" or (lung n1 disease*) or (lung n1 injur*) (34,836)

S28 (MH "Chronic Disease") (45,683)

S27 (MH "Lung Injury+") (7,743)

S26 (MH "Lung Diseases+") (119,975)

S25 (MH "Bronchopulmonary Dysplasia") (1,299)

S24 S22 OR S23 (58,625)

S23 TX "vitamin A" or retinol or retinoid or retinoic or aquasol (58,625)

S22 (MH "Vitamin A") (2,570)

S21 S12 OR S20 (1,524,310)

S20 S13 OR S14 OR S15 OR S16 OR S17 OR S18 OR S19 (514,060)

S19 TX (observational n1 (study or studies)) (41,681)

S18 TX (cohort n1 (study or studies)) (73,791)

S17 (MH "Cross Sectional Studies") (115,355)

S16 (MH "Nonconcurrent Prospective Studies") (180)

S15 (MH "Correlational Studies") (19,933)

S14 (MH "Case Control Studies+") (56,850)

S13 (MH "Prospective Studies") (293,038)

S12 S1 OR S2 OR S3 OR S4 OR S5 OR S6 OR S7 OR S8 OR S9 OR S10 OR S11 (1,157,868)

S11 TX allocat* random* (10,947)

S10 (MH "Quantitative Studies") (15,046)

S9 (MH "Placebos") (9,883)

S8 TX placebo* (73,010)

S7 TX random* allocat* (10,947)

S6 (MH "Random Assignment") (41,906)

S5 TX randomi* control* trial* (163,369)

S4 TX ((singl* n1 blind*) or (singl* n1 mask*) ) or TX ( (doubl* n1 blind*) or (doubl* n1 mask*)) or TX ( (tripl* n1 blind*) or (tripl* n1 mask*) ) or TX ( (trebl* n1 blind*) or (trebl* n1 mask*)) (882,102)

S3 TX clinic* n1 trial* (246,157)

S2 PT Clinical trial (79,809)

S1 (MH "Clinical Trials+") (205,937)
