# Supplementary material for: Vitamin A to prevent bronchopulmonary dysplasia in extremely low birth weight infants: a systematic review and meta-analysis
Source: PLoS One. 2018 Nov 29;13(11):e0207730. doi: 10.1371/journal.pone.0207730 (PMC6264498; doi:10.1371/journal.pone.0207730)
Supplement: S3 Table — (PPTX) [file pone.0207730.s003.pptx]

## Slide 1
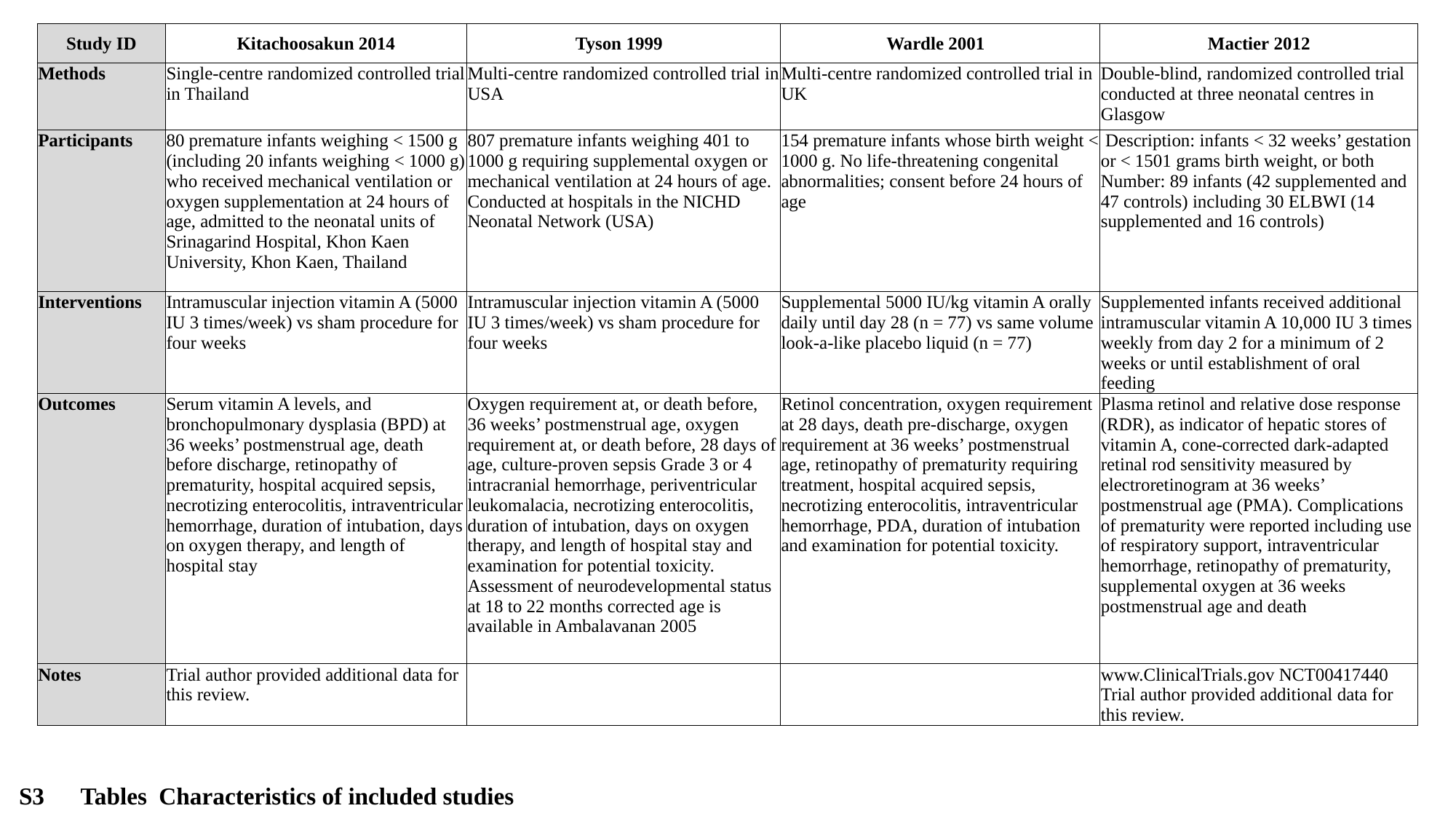

| Study ID | Kitachoosakun 2014 | Tyson 1999 | Wardle 2001 | Mactier 2012 |
| --- | --- | --- | --- | --- |
| Methods | Single-centre randomized controlled trial in Thailand | Multi-centre randomized controlled trial in USA | Multi-centre randomized controlled trial in UK | Double-blind, randomized controlled trial conducted at three neonatal centres in Glasgow |
| Participants | 80 premature infants weighing < 1500 g (including 20 infants weighing < 1000 g) who received mechanical ventilation or oxygen supplementation at 24 hours of age, admitted to the neonatal units of Srinagarind Hospital, Khon Kaen University, Khon Kaen, Thailand | 807 premature infants weighing 401 to 1000 g requiring supplemental oxygen or mechanical ventilation at 24 hours of age. Conducted at hospitals in the NICHD Neonatal Network (USA) | 154 premature infants whose birth weight < 1000 g. No life-threatening congenital abnormalities; consent before 24 hours of age | Description: infants < 32 weeks’ gestation or < 1501 grams birth weight, or both Number: 89 infants (42 supplemented and 47 controls) including 30 ELBWI (14 supplemented and 16 controls) |
| Interventions | Intramuscular injection vitamin A (5000 IU 3 times/week) vs sham procedure for four weeks | Intramuscular injection vitamin A (5000 IU 3 times/week) vs sham procedure for four weeks | Supplemental 5000 IU/kg vitamin A orally daily until day 28 (n = 77) vs same volume look-a-like placebo liquid (n = 77) | Supplemented infants received additional intramuscular vitamin A 10,000 IU 3 times weekly from day 2 for a minimum of 2 weeks or until establishment of oral feeding |
| Outcomes | Serum vitamin A levels, and bronchopulmonary dysplasia (BPD) at 36 weeks’ postmenstrual age, death before discharge, retinopathy of prematurity, hospital acquired sepsis, necrotizing enterocolitis, intraventricular hemorrhage, duration of intubation, days on oxygen therapy, and length of hospital stay | Oxygen requirement at, or death before, 36 weeks’ postmenstrual age, oxygen requirement at, or death before, 28 days of age, culture-proven sepsis Grade 3 or 4 intracranial hemorrhage, periventricular leukomalacia, necrotizing enterocolitis, duration of intubation, days on oxygen therapy, and length of hospital stay and examination for potential toxicity. Assessment of neurodevelopmental status at 18 to 22 months corrected age is available in Ambalavanan 2005 | Retinol concentration, oxygen requirement at 28 days, death pre-discharge, oxygen requirement at 36 weeks’ postmenstrual age, retinopathy of prematurity requiring treatment, hospital acquired sepsis, necrotizing enterocolitis, intraventricular hemorrhage, PDA, duration of intubation and examination for potential toxicity. | Plasma retinol and relative dose response (RDR), as indicator of hepatic stores of vitamin A, cone-corrected dark-adapted retinal rod sensitivity measured by electroretinogram at 36 weeks’ postmenstrual age (PMA). Complications of prematurity were reported including use of respiratory support, intraventricular hemorrhage, retinopathy of prematurity, supplemental oxygen at 36 weeks postmenstrual age and death |
| Notes | Trial author provided additional data for this review. | | | www.ClinicalTrials.gov NCT00417440 Trial author provided additional data for this review. |
S3　Tables Characteristics of included studies
