# Supplementary material for: Vitamin A to prevent bronchopulmonary dysplasia in extremely low birth weight infants: a systematic review and meta-analysis
Source: PLoS One. 2018 Nov 29;13(11):e0207730. doi: 10.1371/journal.pone.0207730 (PMC6264498; doi:10.1371/journal.pone.0207730)
Supplement: S1 Fig — Outcome 5: duration of mechanical ventilation. (PPTX) [file pone.0207730.s004.pptx]

## Slide 1
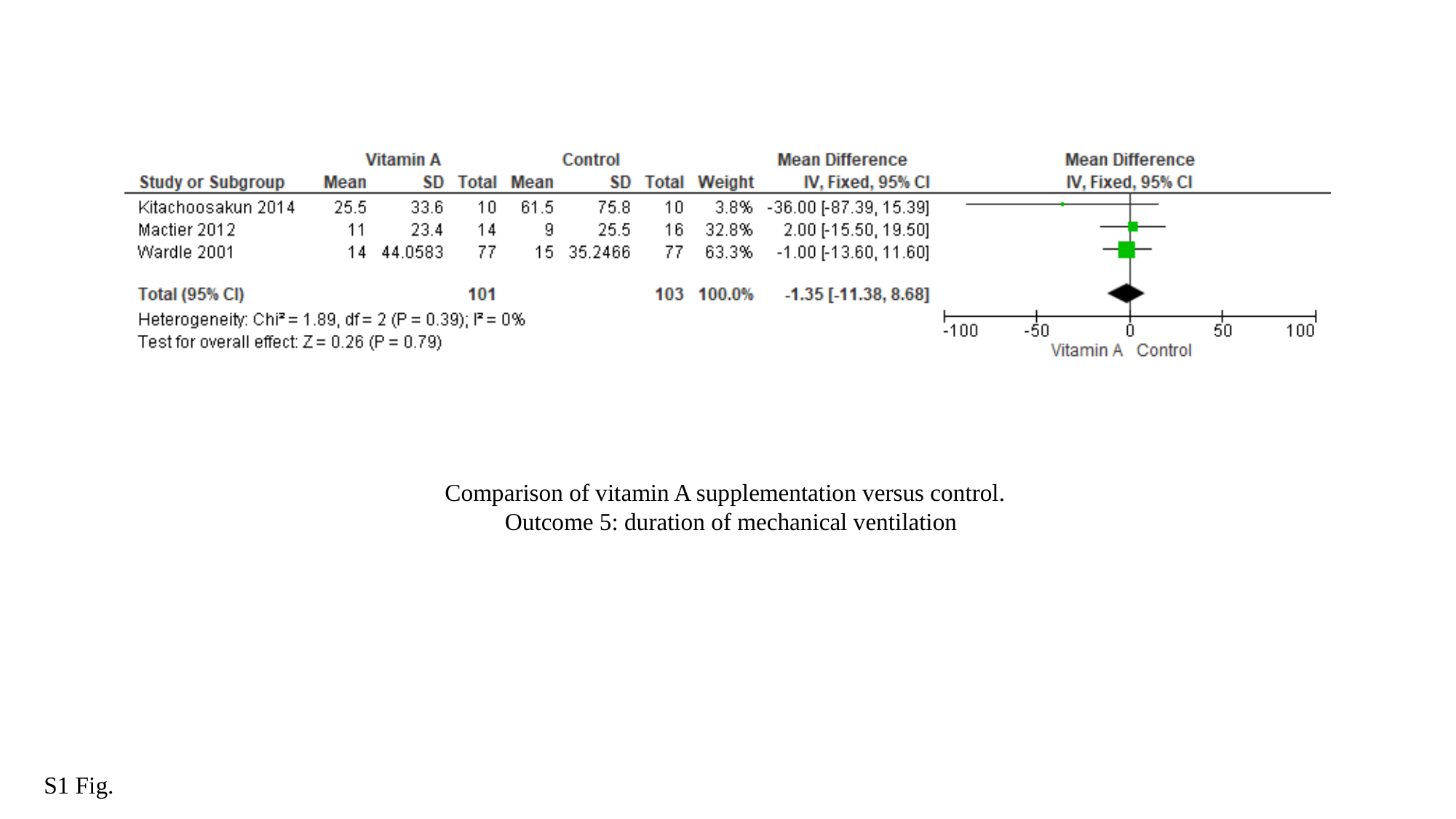

Comparison of vitamin A supplementation versus control.
 Outcome 5: duration of mechanical ventilation
S1 Fig.
